# Status of diagnosis and preventative treatment for primary headache disorders: real-world data of unmet needs in China

**DOI:** 10.1186/s10194-023-01654-6

**Published:** 2023-09-01

**Authors:** Huanxian Liu, Ming Dong, Kaiming Liu, Zhihua Jia, Wei Gui, Yingying Cheng, Yudan Lv, Kang Qu, Hongru Zhao, Jianjun Chen, Dan Zhang, Zhiliang Fan, Xiaosu Yang, Dongmei Hu, Hongyan Xie, Mingxin Li, Bing Wen, Sufen Chen, Peng Xu, Qingqing Rong, Qiu He, Zhanxiu Ren, Fanhong Yan, Heling Zhao, Min Chen, Tingmin Yu, Hongli Qu, Xingkai An, Huailian Guo, Xinhua Zhang, Xiaoping Pan, Xiaojuan Wang, Shi Qiu, Lvming Zhang, Hongling Zhao, Xin Pan, Qi Wan, Lanyun Yan, Jing Liu, Zhe Yu, Mingjie Zhang, Ye Ran, Xun Han, Shengyuan Yu, Zhao Dong

**Affiliations:** 1 Department of Neurology, the First Medical Center of Chinese PLA General Hospital, Beijing, 100853 China; 2 International Headache Center, Chinese PLA General Hospital, Beijing, 100853 China; 3 Department of Neurology and Neuroscience Center, The First Hospital of Jilin University, Jilin, 130021 China; 4 Department of Neurology, Second Affiliated Hospital, School of Medicine, Zhejiang University, Zhejiang, 310009 China; 5 Department of Neurology, The First Affiliated Hospital of USTC, Division of Life Sciences and Medicine, University of Science and Technology of China, Lujiang Road 17, Hefei, 230001 China; 6 Department of Neurology, The First Affiliated Hospital of Soochow University, Jiangsu, 215006 China; 7 Department of Neurology, Lishui Municipal Central Hospital, Zhejiang, 323000 China; 8 Department of Neurology, Sir Run Run Shaw Hospital, Zhejiang, 310020 China; 9 Department of Neurology, Xing Tai People’s Hospital, Hebei, 054001 China; 10 Department of Neurology, Xiangya Hospital, Central South University, Hunan, 410013 China; 11 Department of Neurology, The Second Affiliated Hospital of Shandong First Medical University, Shandong, 271000 China; 12 Department of Neurology, Qilu Hospital, Shandong, 250012 China; 13 Department of Neurology, Changsha Central Hospital Affiliated to the University of South China, Hunan, 410004 China; 14 Department of Neurology, Affiliated Hospital of Jining Medical University, Shandong, 272000 China; 15 Department of Neurology, The People’s Hospital of Liaoning Province, Liaoning, 110016 China; 16 Department of Neurology, Linyi Jinluo Hospital, Shandong, 276036 China; 17 Department of Neurology, Zhengzhou University First Affiliated Hospital, Henan, 450052 China; 18 Department of Neurology, The Second Hospital of Jilin University, Jilin, 130041 China; 19 Department of Neurology, The First Affiliated Hospital of Xiamen University, Jilin, 130041 China; 20 Department of Neurology, People’s Hospital, Peking University, Beijing, 100044 China; 21 Department of Neurology, Guangzhou First People’s Hospital, Guangdong, 510180 China; 22 Department of Neurology, Aerospace Center Hospital, Beijing, 100049 China; 23 Department of Neurology, Da Lian Municipal Central Hospital, Liaoning, 116033 China; 24 Department of Neurology, Jiangsu Province Hospital, Jiangsu, 210029 China

**Keywords:** Diagnosis, Treatment, Primary headache disorders, Migraine, Tension-type headache

## Abstract

**Background:**

Headache disorders are widely prevalent and pose a considerable economic burden on individuals and society. Globally, misdiagnosis and inadequate treatment of primary headache disorders remain significant challenges, impeding the effective management of such conditions. Despite advancements in headache management over the last decade, a need for comprehensive evaluations of the status of primary headache disorders in China regarding diagnosis and preventative treatments persists.

**Methods:**

In the present study, we analyzed the established queries in the Survey of Fibromyalgia Comorbidity with Headache (SEARCH), focusing on previous diagnoses and preventative treatment regimens for primary headache disorders. This cross-sectional study encompassed adults diagnosed with primary headache disorders who sought treatment at 23 hospitals across China between September 2020 to May 2021.

**Results:**

The study comprised 2,868 participants who were systematically examined. Migraine and tension-type headaches (TTH) constituted a majority of the primary headache disorders, accounting for 74.1% (2,124/2,868) and 23.3% (668/2,868) of the participants, respectively. Medication overuse headache (MOH) affected 8.1% (231/2,868) of individuals with primary headache disorders. Over half of the individuals with primary headache disorders (56.6%, 1,624/2,868) remained undiagnosed. The previously correct diagnosis rates for migraine, TTH, TACs, and MOH were 27.3% (580/2,124), 8.1% (54/668), 23.2% (13/56), and 3.5% (8/231), respectively. The misdiagnosis of “Nervous headache” was found to be the most prevalent among individuals with migraine (9.9%, 211/2,124), TTH (10.0%, 67/668), trigeminal autonomic cephalalgias (TACs) (17.9%, 10/56), and other primary headache disorders (10.0%, 2/20) respectively. Only a minor proportion of individuals with migraine (16.5%, 77/468) and TTH (4.7%, 2/43) had received preventive medication before participating in the study.

**Conclusions:**

While there has been progress made in the rate of correct diagnosis of primary headache disorders in China compared to a decade ago, the prevalence of misdiagnosis and inadequate treatment of primary headaches remains a veritable issue. As such, focused efforts are essential to augment the diagnosis and preventive treatment measures related to primary headache disorders in the future.

**Supplementary Information:**

The online version contains supplementary material available at 10.1186/s10194-023-01654-6.

## Introduction

Primary headache disorders, which include migraine, tension-type headaches (TTH), trigeminal autonomic headaches (TACs), including cluster headaches, and other primary headaches disorders, pose a significant health concern [1]. Headache disorders, particularly migraine, TTH, and medication overuse headaches (MOH), are highly prevalent worldwide, and have the potential to cause significant disability, reduced quality of life, impaired productivity, as well as a significant economic burden on both individuals and society [2]. Correct diagnosis and appropriate treatment are critical in reducing the detrimental impacts of headache disorders on patients. However, misdiagnosis and inadequate treatment of primary headache disorders persist as considerable challenges [3].

In China, a population-based study unveiled that a significant proportion of patients with migraine or TTH had not received a correct diagnosis previously, with correct diagnosis rates of 13.8% and 5.6%, respectively [4, 5]. Similarly, a clinic-based study from the Chinese mainland indicated that just 13.5% of individuals with migraine received a physician’s diagnosis of migraine [6]. Moreover, merely 2.7% of these individuals had been prescribed preventative medications [6]. Many attempts have been made during the past decade to improve the diagnosis and clinical management of headaches in China, including the establishment of more than 200 headache clinics throughout the country, translation into Chinese of the International Classification of Headache Disorders (ICHD), 2nd edition (ICHD-2) and ICHD-3, publication of guidelines for the diagnosis and clinical management of primary headache disorders, and provision of fundamental training to primary care personnel [7, 8]. However, the effectiveness of these initiatives have not been reevaluated concerning correct diagnosis and preventive treatment of primary headache disorders.

Therefore, we built an inquiry into our recent Survey of Fibromyalgia Comorbidity with Headache (SEARCH) study, designed to assess fibromyalgia's prevalence and clinical features in Chinese hospital patients with primary headache disorders. The additional survey reported here primarily assesses the status of diagnosis and preventative treatment of primary headache disorders in China.

## Methods

### Study population

This cross-sectional study was a secondary analysis of data from the SEARCH study, the details of which have been described previously [9]. Approval was obtained from the Medical Ethics Committee of Chinese PLA General Hospital (approval number: S2020-238–01), and the study was registered with the Chinese Clinical Trial Registry, with registration number ChiCTR2000034894. Written informed consent was obtained from all participants. Chief complaints of patients during consultation formed the basis of diagnosis for primary headache disorders. Inclusion criteria were consistent with the SEARCH study [9]. Unlike the SEARCH study, patients who had previously used fibromyalgia treatment medication or had incomplete information about their fibromyalgia diagnosis were not excluded from this study. However, participants with missing data in previous diagnosis or prevention history were excluded.

Uniform Questionnaires were used to collect data on sociodemographics (gender, age, race, educational level, individual income, and body mass index), headache characteristics, anxiety, depression, and sleep, in addition to deliberately asking about previous headache diagnosis and prevention treatment. Primary headache disorders were diagnosed according to the ICHD-3 [1]. Detailed information about the questionnaire, and the definition of the variables has been described previously [9]. We classified previous headache diagnoses as “undiagnosed”, “migraine”, “tension-type headache”, “cluster headache”, “vascular headache”, “nervous headache”, or “other” based on the classification of previous headache diagnoses in a previous study [4]. We also explored the previous preventative treatment of patients with the correct diagnosis.

### Analysis of baseline characteristics

Analysis was performed using R 4.2.1 (http://www.R-project.org; The R Foundation, Vienna, Austria) and the Free Statistics software (version 1.8; Beijing FreeClinical Medical Technology Co., Ltd, Beijing, China) [10]. Descriptive statistics include mean (standard deviation), median (interquartile range), and frequencies (percentage) as appropriate. We describe the essential characteristics of the overall population and the different primary headache types. In addition, we compared the distribution of comorbidities between groups with delayed diagnosis of primary headache disorders. Differences between the two groups were compared using the chi-square test.

## Results

**Fig. 1:**
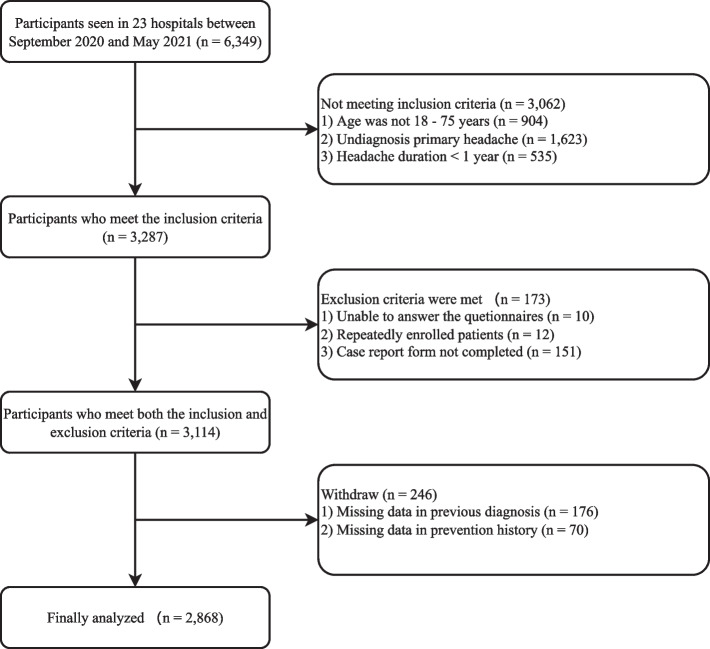
Flow chart

Of the 6,349 patients screened in 23 headache centers, 3,062 did not meet the inclusion criteria, 173 met the exclusion criteria, and 246 lacked data on previous diagnoses (*n* = 176) and prevention history (*n* = 70). Finally, 2,868 participants were included in the final analyses (Fig. 1).

The characteristics of the participants are shown in Table 1. The mean age was 42.1 (12.9) years, and 2,101 (73.3%) were women. 97.6% (2,799/2,868) of the patients were Han Chinese. Regarding educational level, 1,286 (44.8%) participants had a high school or lower. The median headache duration was 8.0 (3.0, 15.0) years. Regarding headache family history, 1,118 (44.8%) participants had a headache family history. The number of patients with combined anxiety, depression, insomnia, and fibromyalgia were 101 (3.5%), 345 (12.0%), 583 (20.3%), and 157 (5.5%), respectively.

**Table 1:** Characteristics of individuals with primary headache

Variables	Total (*n* = 2,868)	Migraine (*n* = 2,124)	TTH (*n* = 668)	TACs (*n* = 56)	Others (*n* = 20)
**Gender, n (%)**					
Man	767 (26.7)	484 (22.8)	232 (34.7)	43 (76.8)	8 (40)
Woman	2,101 (73.3)	1,640 (77.2)	436 (65.3)	13 (23.2)	12 (60)
**Age, years, Mean (SD)**	42.1 (12.9)	40.9 (12.3)	46.2 (13.8)	36.6 (10.8)	43.4 (12.2)
**Race**^**a**^**, n (%)**					
Han	2,799 (97.6)	2067 (97.3)	658 (98.5)	54 (96.4)	20 (100)
Other	66 (2.3)	54 (2.5)	10 (1.5)	2 (3.6)	0 (0)
**Educational level**^**b**^**, n (%)**					
High school	1,286 (44.8)	902 (42.5)	344 (51.5)	26 (46.4)	14 (70)
> High school	1367 (47.7)	1079 (50.8)	256 (38.3)	26 (46.4)	6 (30)
**Income per month**^**c**^**, n (%)**					
< ¥ 3000	311 (10.8)	214 (10.1)	92 (13.8)	1 (1.8)	4 (20)
[¥ 3000, ¥ 6000)	437 (15.2)	302 (14.2)	121 (18.1)	10 (17.9)	4 (20)
[¥ 6000, ¥ 9000]	238 (8.3)	177 (8.3)	51 (7.6)	8 (14.3)	2 (10)
> ¥ 9000	233 (8.1)	166 (7.8)	57 (8.5)	6 (10.7)	4 (20)
**BMI, kg/m**^2^**, Mean (SD)**	24.4 (4.7)	24.2 (4.7)	25.0 (4.6)	25.0 (3.5)	25.1 (3.7)
**Headache duration, years, Mean (SD)**	10.0 (8.7)	10.9 (8.9)	7.5 (7.7)	7.7 (5.4)	4.2 (3.6)
**VAS, Mean (SD)**	6.3 (1.9)	6.7 (1.7)	4.9 (1.8)	8.1 (1.6)	5.1 (1.8)
**Headache family history, n (%)**	1,118 (39.0)	948 (44.6)	154 (23.1)	16 (28.6)	0
**MOH, n (%)**	231 (8.1)	193 (9.1)	38 (5.7)	0	0
**Anxiety**^**d**^**, n (%)**	101 (3.5)	73 (3.4)	27 (4.0)	1 (1.8)	0
**Depression**^**d**^**, n (%)**	345 (12.0)	248 (11.7)	90 (13.5)	5 (8.9)	2 (10.0)
**Insomnia**^**e**^**, n (%)**	583 (20.3)	430 (20.2)	134 (20.1)	14 (25.0)	5 (25.0)
**Fibromyalgia, n (%)**	157 (5.5%)	112 (5.3%)	43 (6.4)	1 (1.8)	1 (5%)

*Abbreviations*: *TTH* Tension-type headache, *TACs* Trigeminal autonomic cephalalgias, *BMI* Body mass index, *VAS* Visual analog scale, *MOH* Medication overuse headache

^a^Data are missing for 3 (0.1%) patients

^b^Data are missing for 215 (7.5%) patients

^c^Data are missing for 1649 (57.5%) patients

^d^Data are missing for one patient

^e^Data are missing for 3 (0.1%) patients

**Fig. 2:**
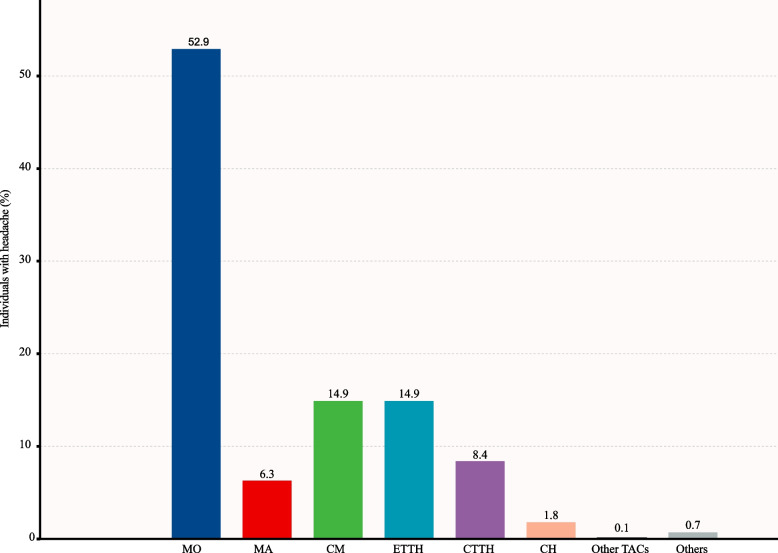
The proportion of different primary headache types. Other trigeminal autonomic cephalalgias (TACs) included short-lasting unilateral neuralgiform headache attacks with conjunctival injection and tearing and hemicrania continua. The residual category “Others” included primary cough headache, primary headache associated with sexual activity, primary stabbing headache, cold-stimulus headache, nummular headache, and new daily persistent headache. MO, migraine without aura; MA, migraine with aura; CM, chronic migraine; ETTH, episodic tension-type headache; CTTH, chronic tension-type headache; CH, cluster headache

Figure 2 displays the distribution of primary headache disorders. The most prevalent diagnoses were migraine without aura, which accounted for 52.9% (1,516/2,868), followed by chronic migraine (14.9%, 427/2,868), episodic and chronic tension-type headaches (14.9% [427/2,868] and 8.4% [241/2,868] respectively), migraine with aura (6.3%, 181/2,868), cluster headache (1.8%, 52/2,868), other primary headaches (0.7%, 20/2,868), and other TACs (0.1%, 4/2,868). In terms of other primary headache disorders (*n* = 20), primary stabbing headache was the most common (*n* = 14), with the remaining diagnoses being primary headaches associated with sexual activity (*n* = 1), cough (*n* = 1), cold stimuli (*n* = 1), nummular headaches (*n* = 1), and new daily persistent headaches (*n* = 2). Among the other TACs (*n* = 4), we found short-lasting unilateral neuralgiform headache attacks with conjunctival injection and tearing (*n* = 3) and hemicrania continua (*n* = 1).

**Fig. 3:**
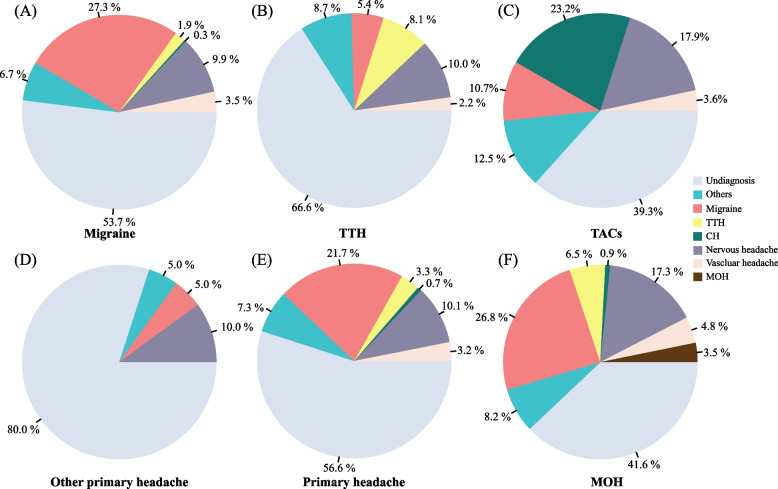
Percentage of previous diagnoses of primary headache disorders and MOH. The cumulative percentage was over 100% due to a patient’s multiple previous diagnoses. TTH, tension-type headache; TACs: trigeminal autonomic cephalalgias; CH, cluster headache; MOH: medication overuse headache

The proportion of different previous headache diagnoses is shown in Fig. 3. 56.6% (1,624/2,868) of individuals with primary headache disorders were undiagnosed (Fig. 3E). Specifically, 53.7% (1,141/2,124) of individuals with migraine (Fig. 3A), 66.6% (445/668) of TTH patients (Fig. 3B), 39.3% (22/56) of TACs patients (Fig. 3C), 80% (16/20) of patients with other primary headaches (Fig. 3D), and 41.6% (96/231) of MOH patients (Fig. 3F) were undiagnosed. “Nervous headache” was the most common misdiagnosis among patients with migraine (9.9%, 211/2,124), TTH (10.0%, 67/668), TACs (17.9%, 10/56), and other primary headache disorders (10.0%, 2/20). Of the 231 patients with MOH, 8 individuals (3.5%, 8/231) had a previous diagnosis of MOH. Among those pre-existing primary headache disorders, 31.1% (60/193) of patients were correctly diagnosed with migraine, and 5.3% (2/38) of patients were correctly diagnosed with TTH. Overall, 28.6% of MOH patients had previously been correctly diagnosed with migraine, TTH, or MOH. The previously reported correct diagnosis for migraine, TTH, and TACs was 27.3% (580/2,124), 8.1% (54/668), and 23.2% (13/56), respectively. In patients with TACs, only some cluster headaches (13/52) were correctly diagnosed, while the other’s TACs were not.

The proportion of comorbid anxiety (3.5% vs. 3.6%, *p* = 0.960), depression (12.3% vs. 11.0%, *p* = 0.346), insomnia (20.7% vs. 19.0%, *p* = 0.348), and fibromyalgia (5.4% vs. 5.6%, *p* = 0.909) were similar between the delayed diagnosis group and the non-delayed group (Table S1).

**Fig. 4:**
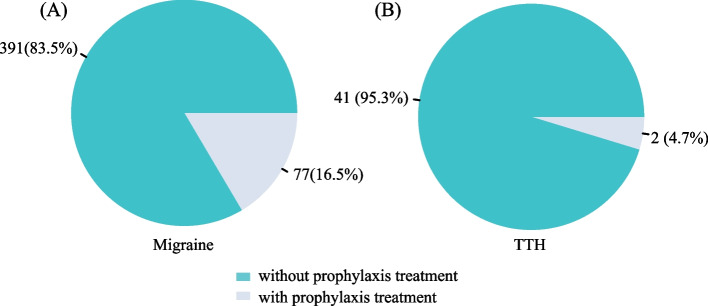
Percentage of previous prophylaxis treatment of migraine (A) and TTH (B). TTH, tension-type headache

**Fig. 5:**
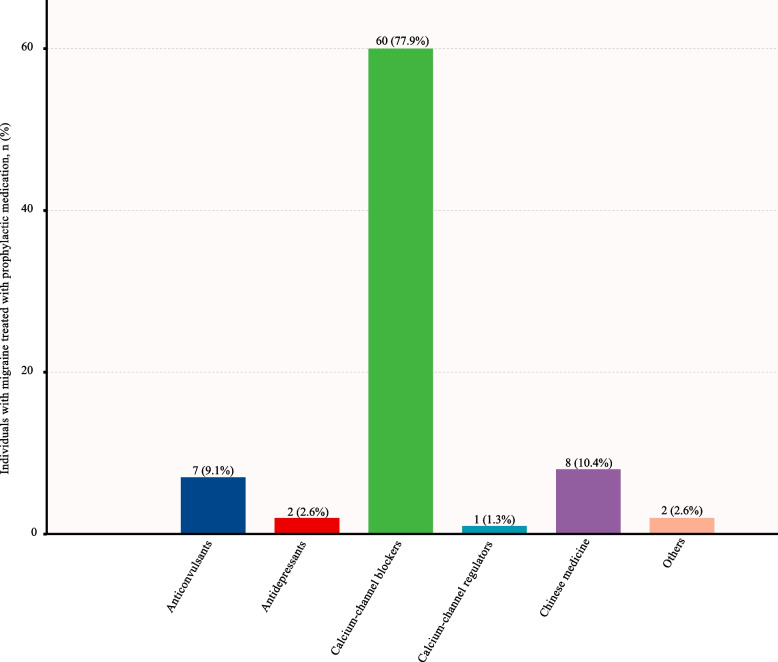
Prophylactic medications previously used by individuals with migraine. The cumulative percentage exceeded 100% due to a patient's previous use of multiple preventative drugs

Figure 4 illustrates the prophylactic treatment status of patients previously correctly diagnosed with migraine and TTH. Among the 468 participants with an affirmed medical diagnosis of migraine, only 16.5% (77/468) had taken prophylactic medication previously (Fig. 4A). The most frequently used preventative treatment was calcium-channel blockers, which were used by 77.9% (60/77) of the patients (Fig. 5). Out of the 43 patients accurately diagnosed with TTH, only two of them (4.7%) received prophylactic treatment with antidepressants (Fig. 4B). Even though four patients with TACs were accurately diagnosed, none received preventive medicine. Similarly, only one of the eight patients correctly diagnosed with MOH was prescribed prophylactic medication.

## Discussion

The rate of correct diagnosis for migraine, TTH, TACs, and MOH recorded previously stood at 27.3%, 8.1%, 23.2%, and 3.5%, respectively. Only 16.5% of individuals accurately diagnosed with migraine and 4.7% of those accurately diagnosed with TTH received prophylactic medication included in the treatment regimen.

The proportion of patients admitted to the hospital outpatient with primary headache disorders shifted regarding the headache type compared to a decade ago. Compared to previous studies in China, migraine disorders increased to 74.1% from 47.6%-49.9%, while the proportion of TTH decreased to 23.3% from 41.5%-47.3% previously [11, 12], suggesting that migraine accounts for most patients presenting to the hospital with primary headache disorders. Similarly, a recent hospital-based study of headache types among headache patients attending outpatient clinics in the Middle East, Asia, and Africa reported that many individuals experienced migraine (57%) compared to TTH patients (32.2%) [13]. The precise cause for the changes in the proportion of individuals with migraine seeking treatment in outpatient clinics remains unclear. We speculate that several factors may have contributed to this shift. One of these factors is the establishment of 31 local headache centers and over 200 headache clinics in China, which have introduced a new era of personalized care for patients suffering from headaches [7]. In addition, to ensure that healthcare professionals are well-informed of the latest advances in the field, comprehensive training courses and headache conferences have been held in major cities across the country, aimed at providing headache specialists and medical students with the most up-to-date knowledge on all aspects of headache disorders [7]. It is our belief that by implementing these measures, recognition of headache disorders by doctors in China can be improved. Secondly, migraine disorders are typically more severe than TTH, which may encourage individuals with migraine to seek hospital treatment more frequently, thereby explaining why there are more patients with migraine disorders among primary headache patients seen in hospital [14]. Additionally, the current study reported an increase in the correct diagnosis rate for both migraine (from 13.8% to 27.3%) and TTH (from 5.6% to 8.1%) compared to the previous diagnosis rate [4]. However, the increase in correct diagnosis rate was more apparent for migraine than TTH. This suggests that an increased number of individuals with migraine may have been identified as a result of improved diagnostic accuracy. In contrast, TTH may not have received the same benefit from the improvements in diagnostic accuracy, which may have contributed to the observed higher proportion of people with migraine when compared to TTH in the study population.

Misdiagnosis of primary headache disorders continues to influence patients globally (Table 2). Despite an increase in the proportion of accurately diagnosed migraine disorders in China from 13.8% to 27.3% and TTH from 5.6% to 8.1%, the overall correct diagnosis rate remains low [4]. Comparable diagnostic failures of primary headache disorders also occur in other countries. A transcontinental study involving individuals with migraine disorders referred to specialist headache centers showed that general practitioners misdiagnosed 72% of patients with "headaches" (64%), "neck pain" (4%), "tension headache" (3%), and sinusitis (1%) [15]. A secondary analysis of the population-representative 2009 American Migraine Prevalence and Prevention study revealed that 45.5% of individuals with migraine received medical consultation within a year, while 86.7% of the respondents received an accurate migraine diagnosis [16]. A decade later, the OVERCOME 2018 migraine cohort study in the US found that 61.0% of individuals with migraine disorders self-reported receiving a medical diagnosis of migraine [17]. In Russia, only 12% of people with migraine and 11.7% of individuals with TTH received a correct diagnosis [18]. Common misdiagnoses included autonomic vascular dysfunctions (56%), cervical osteochondrosis (35%), and intracranial hypertension (10%) [18]. In Japan, a mere 11.6% of participants experiencing migraine were aware that they had migraine [19]. It is estimated that only 13% of individuals with migraine in low-income countries are diagnosed by healthcare professionals [20]. Moreover, this study also provides insights into the previously recorded correct diagnoses of TACs and MOH. The results of this study regarding the prior accurate diagnosis of MOH (3.5%) are in line with an earlier hospital-based study, which reported that very few patients with MOH, 0.8% (2/240), had received a previously correctly diagnosed [21]. When taken together, the total rate of correct diagnosis in primary headache patients and MOH receiving health-care continues to be unfavorable, indicating the need for additional measures to improve it.

**Table 2:** Studies report previous diagnoses and preventative treatment of primary headache disorders

Citation	Country/region	Study design/study dates	Criteria	Population (n)	Main findings
Liu R, et al. [4]	China	cross-sectional population-based/2009	ICHD-2	452 individuals with migraine and 531 patients with TTH	1.13.8% of individuals with migraine and 5.6% of patients with TTH were correctly diagnosed previously
Katsarava Z, et al. [23]	10 Europe countries	cross-sectional population-based/2009	ICHD-2	3466 participants with migraine	1.3.1%-18.0% of individuals with migraine have never seen a doctor 2.1.6%-13.7% of eligible patients in the population-based sample used migraine-preventative medication
Lipton RB, et al. [16]	US	cross-sectional population-based/2009	ICHD-2	775 individuals with migraine	1.45.5% of individuals with migraine disorders consulted a health professional in the past year, and 86.7% were diagnosed with migraine
Li X, et al. [6]	China	cross-sectional neurological clinic/2010	ICHD-2	401 individuals with migraine	1.13.5% of individuals with migraine had received a physician diagnosis of migraine 2. Only 11 (2.7%) individuals with migraine had used preventative medications
Lebedeva ER, et al. [18]	Russia	cross-sectional/2012–2013	ICHD-3β	1042 students, 1075 workers, and 1007 blood donors	1.12% of the patients with migraine and 11.7% of patients with TTH had previously been correctly diagnosed 2.0.4% of individuals with migraine and no TTH patients had received prophylactic treatment
Viana M, et al. [15]	Seven countries	multicentre study/2015–2016	ICHD-3β	1161 individuals with migraine	1. General practitioners misdiagnosed 72% of individuals with migraine 2.29% of patients received at least one migraine preventive medication
Lipton RB, et al. [17]	US	cross-sectional population-based/2018	ICHD-3	19,888 individuals with migraine	1.12,905 (61.0%) self-reported a medical diagnosis of migraine 2. 41.6% of those eligible for migraine preventive treatment currently use migraine preventive medication
Takeshima T, et al. [19]	China, Japan, and South Korea	review/2019	IHS ICHD-2 ICHD-3β	migraine	1. In China, 13.5% to 18% of individuals with migraine had been diagnosed with migraine previously 2. In Japan, 11.6% of individuals with migraine were aware that they had migraine 3. There was limited information in South Korea
Kim BK, et al. [24]	South Korea	multicentre study/2019	ICHD	207 participants with migraine	1. Only 23.7% of individuals with migraine had regularly taken preventive medication

*ICHD* International Classification of Headache Disorders, *IHS* International Headache Society, *TTH* tension-type headache

Besides misdiagnosis, inadequate preventive treatment for patients with primary headache disorders remains another prevalent concern (Table 2). Our research identified that prior utilization of preventive medication by those with migraine and TTH was recorded at 16.5% and 4.7%, respectively. In comparison, another report from a clinical setting indicated the usage to be only 2.7% of individuals with migraine [6]. In Chinese Taiwan, 40.2% of migraine outpatients reported using preventive medicines [22]. Correspondingly, the Eurolight study -a cross-sectional questionnaire survey- conducted in ten European countries uncovered that only 1.6–13.7% of eligible individuals in a population-based European sample utilized preventative medication for migraine disorders [23]. Likewise, in Russia, prophylactic medication was used by only 0.4% of individuals with migraine and zero TTH patients [18]. The OVERCOME study findings in the US stated that although eligibility, 41.6% of those utilizing migraine preventive medication, was revealed [17]. For South Korea, only 23.7% of individuals with migraine disorders reportedly used preventive medication regularly [24]. Taken together, it can be concluded that the prevalence of preventive medication use remains low among people with migraine worldwide, even in countries with high-income levels [23].

A literature review with meta-analysis has shown that patients with primary headache disorders are often comorbid with depression, anxiety, sleep disorders, and fibromyalgia, and this finding was also observed in the present study [25]. 5.5% of individuals with migraine had comorbid fibromyalgia in our study, which is consistent with the findings (5.09%) of Patel et al. [26]. Additionally, previous study demonstrated that migraine is a triggering factor for fibromyalgia, suggesting that in individuals with migraine we should screen for the co-existence of fibromyalgia [27]. Overall, it is the fact that primary headache sufferers are prone to comorbidities that makes it even more challenging to properly diagnose primary headache patients, which can be read as a litmus test of the ability to correctly diagnose primary headaches.

This study exhibits multiple noteworthy strengths, including its large sample size and multicenter design. Additionally, the ICHD-3 version was adapted to aid in diagnosing primary headache disorders. Nevertheless, several limitations require consideration. Firstly, the exclusive study of hospital patients may lead to biased results and limited generalizability. Secondly, diagnosis focusing on the most troublesome headache features may neglect to co-occur primary headache disorders. Thirdly, the obtained data regarding previous diagnoses and preventative treatments were participants to self-reporting, which can lead to recall bias. Fourth, we did not analysis further the factors affecting delayed diagnosis and the duration of delayed diagnosis. The headache questionnaire used in the SEARCH study was a reference to previous questionnaires investigating the burden of headache in China [4, 5, 28]. Moreover, the SEARCH study was not specifically designed to address the current state of primary headache disorders care in China, and therefore did not collect information that affects the correct diagnosis of primary headache and the duration of diagnostic delay. Finally, the classification of previous headache diagnoses in this study was not based on the International Headache Classification, but on the diagnostic classification used in previous study in China [4]. Therefore, the classification of previous headache diagnoses cannot be generalized to other countries.

## Conclusions

Our study’s findings indicate that primary headache disorders are frequently misdiagnosed and inadequately treated, pointing to insufficient use of ICHD guidelines and other diagnostic and clinical management protocols in China. These results highlight the pressing need for sustained campaigns and educational efforts worldwide to improve the diagnosis and treatment of primary headache disorders.

### Supplementary Information

**Additional file 1:** **Table S1.** Distribution of comorbidities in the diagnostic delay group in primary headache disorders.

## Data Availability

The study data are available and may be shared upon reasonable request to the corresponding authors.

## Abbreviations

TTH: Tension-type headaches
TACs: Trigeminal autonomic headaches
MOH: Medication overuse headaches
ICHD: International classification of headache disorders
SEARCH: Survey of fibromyalgia comorbidity with headache
