# Supplementary material for: Status of diagnosis and preventative treatment for primary headache disorders: real-world data of unmet needs in China
Source: J Headache Pain. 2023 Sep 1;24(1):119. doi: 10.1186/s10194-023-01654-6 (PMC10472552; doi:10.1186/s10194-023-01654-6)
Supplement: Supplementary file 1 — Additional file 1: Table S1. Distribution of comorbidities in the diagnostic delay group in primary headache disorders. [file 10194_2023_1654_MOESM1_ESM.docx]

Table S1. Distribution of comorbidities in the diagnostic delay group in primary headache disorders.

| **Variables** | Total  (n = 2,868) | Diagnostic delay | | ***p*-value** |
| --- | --- | --- | --- | --- |
|  |  | No  (n = 647) | Yes  (n = 2,211) |  |
| **Anxiety ^a^, n (%)** |  |  |  | 0.960 |
| No | 2,766 (96.5) | 624 (96.4) | 2,142 (96.4) |  |
| Yes | 101 (3.5) | 23 (3.6) | 78 (3.5) |  |
| **Depression ^a^, n (%)** | |  |  | 0.346 |
| No | 2,522 (88.0) | 576 (89.0) | 1,946 (87.6) |  |
| Yes | 345 (12.0) | 71 (11.0) | 274 (12.3) |  |
| **Insomnia ^b^, n (%)** |  |  |  | 0.348 |
| No | 2,282 (79.7) | 523 (80.8) | 1,759 (79.2) |  |
| Yes | 583 (20.3) | 123 (19.0) | 460 (20.7) |  |
| Fibromyalgia, n (%) |  |  |  | 0.909 |
| No | 2,711 (94.5) | 611 (94.4) | 2,100 (94.6) |  |
| Yes | 157 (5.5) | 36 (5.6) | 121 (5.4) |  |

^a^ Data are missing for one patient.

^b^ Data are missing for 3 (0.1%) patients.
